# Implementing and Measuring the Level of Laboratory Service Integration in a Program Setting in Nigeria

**DOI:** 10.1371/journal.pone.0107277

**Published:** 2014-09-11

**Authors:** Henry Mbah, Olubunmi Ruth Negedu-Momoh, Oluwasanmi Adedokun, Patrick Anibbe Ikani, Oluseyi Balogun, Olusola Sanwo, Kingsley Ochei, Maurice Ekanem, Kwasi Torpey

**Affiliations:** Family Health International (FHI-360), Garki-Abuja, Nigeria; Tulane University School of Public Health and Tropical Medicine, United States of America

## Abstract

**Background:**

The surge of donor funds to fight HIV&AIDS epidemic inadvertently resulted in the setup of laboratories as parallel structures to rapidly respond to the identified need. However these parallel structures are a threat to the existing fragile laboratory systems. Laboratory service integration is critical to remedy this situation. This paper describes an approach to quantitatively measure and track integration of HIV-related laboratory services into the mainstream laboratory services and highlight some key intervention steps taken, to enhance service integration.

**Method:**

A quantitative before-and-after study conducted in 122 Family Health International (FHI360) supported health facilities across Nigeria. A minimum service package was identified including management structure; trainings; equipment utilization and maintenance; information, commodity and quality management for laboratory integration. A check list was used to assess facilities at baseline and 3 months follow-up. Level of integration was assessed on an ordinal scale (0 = no integration, 1 = partial integration, 2 = full integration) for each service package. A composite score grading expressed as a percentage of total obtainable score of 14 was defined and used to classify facilities (≤80% FULL, 25% to 79% PARTIAL and <25% NO integration). Weaknesses were noted and addressed.

**Results:**

We analyzed 9 (7.4%) primary, 104 (85.2%) secondary and 9 (7.4%) tertiary level facilities. There were statistically significant differences in integration levels between baseline and 3 months follow-up period (p<0.01). Baseline median total integration score was 4 (IQR 3 to 5) compared to 7 (IQR 4 to 9) at 3 months follow-up (p = 0.000). Partial and fully integrated laboratory systems were 64 (52.5%) and 0 (0.0%) at baseline, compared to 100 (82.0%) and 3 (2.4%) respectively at 3 months follow-up (p = 0.000).

**Discussion:**

This project showcases our novel approach to measure the status of each laboratory on the integration continuum.

## Introduction

Despite the pivotal role of laboratory services in the health care system, it has been grossly neglected in Africa over the decades [1]_,_ [2]_._ From 2004 to 2012, Family Health International (FHI360) with funding from the President’s Emergency Plan for AIDS Relief (PEPFAR) through USAID and Global Fund to fight AIDS TB and Malaria (GFATM) supported the Government of Nigeria to strengthen 134 laboratories to provide antiretroviral treatment (ART) services for the management of people living with HIV&AIDS (PLWHA). However, because of the emergency approach of early phase of PEPFAR to save lives by putting as many HIV infected persons on ART, these laboratories were set up to fight HIV&AIDS as a single disease entity. The unintended effects of this approach was the establishment of parallel and competing structures which actually threatened the existing precarious laboratory system [3]. In the field, it is common to see a newly renovated, equipped and well-staffed HIV laboratory side-by-side with a crumbling general facility laboratory. In the absence of donor funds, sustaining these supported laboratories remain a challenge. In line with PEPFAR II goals to improve the quality of service, ensure ownership and sustainability, laboratory service integration is warranted. In developing countries, the integration of public health laboratories within clinical programs serving both those with HIV and the general public is essential [3]. These laboratories can be strengthened in a sustainable way by leveraging from funding from other sources in addition to HIV&AIDS prevention, care, surveillance, and treatment programs [4]. Parsons et al [5] described an integrated laboratory service as a laboratory that is capable of providing all primary diagnostic services needed for the care and treatment of patients without requiring different laboratories for specific tests.

Service integration is a mechanism for organizing and blending interrelated health issues, activities, and prevention strategies to facilitate comprehensive delivery of services [6]. A key benefit of service integration is that it encourages service providers to offer various interrelated services to persons whenever they access services. Furthermore, healthcare providers can achieve target outcomes more easily with less investment by coordinating available, necessary, and preferable human services to patients [7]. Sweeney et al in a recent study concluded that integration of HIV services with other health services are cost-effective and efficient [8]. However critics of health service integration argue that it is not a cure for inadequate resources [9] and leads to dilution of expertise and degrade quality of service in areas where the health system is weak particularly in terms of logistical challenges and health workforce shortages [10], [11], [12]. Integration of services generates demand for an increased pool of skilled health workers, which requires both additional resources and also a significant amount of time for training [12], [13].

There is paucity of published information that describes the integration of HIV-related laboratory service into the mainstream laboratory aiming at leveraging resources and improving quality of service. The integration of other health services such as TB/HIV, [14] family planning/RH/HIV [15], [16], [17] HIV/Antenatal [18], [19] or specific services like HIV Care with Primary Health Care Services [20], [21] have been described. As health service integration becomes a dominant programmatic approach to patient care, a demand for integrated laboratory services becomes compelling [6].

Laboratory service integration may be achieved in our current laboratory setup through the use of same equipment, common information and logistics management system, same personnel and administrative structure. However, because of the current status of laboratory settings, there is no “one size fits all” approach to laboratory service integration. Different types of integration approach may be appropriate for different health care facilities or programs, depending on available resources, capacity, and facility set-up [22]. Any approach must take into consideration existing capabilities, buy-in from the facilities personnel and clients they serve, for it to be acceptable and sustainable. FHI360 Nigeria’s overall goal to promote laboratory service integration is to ensure that any investment to strengthen the HIV related laboratory services should cut across all the disease entities to benefit the entire patient community as well as the various health specialties.

Integrated healthcare delivery is a policy goal of healthcare systems. There is no consensus on how to measure the concept, which makes it difficult to monitor progress [23], [24]. There is a need to establish quantitative baseline estimates of current levels of laboratory service integration, and identify areas with gaps to be strengthened. Here we describe an approach to quantitatively measure and classify the level of laboratory service integration and highlight some key intervention steps taken, to enhance laboratory services integration.

## Methods

### Study design & Sites

**Figure 1:**
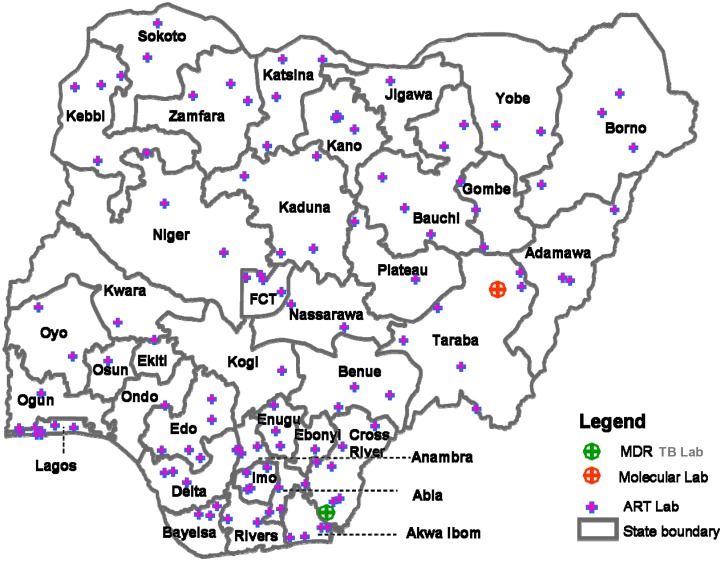
Study Sites: A wide network of FHI360 supported laboratories in Nigeria.

This was a quantitative and before-and-after health facility based study looking at the level of laboratory service integration. The study was conducted in 122 FHI360 supported facilities covering all the 36 states in Nigeria including the Federal Capital Territory (FCT) (Figure 1).

### Approach & definitions

A concept paper was drafted detailing guidance to the study using two approaches of measuring laboratory service integration namely; physical/structural integration and virtual/service integration. These approaches are defined below for purpose of implementation.

#### Physical/Structural Integration

This is combining the infrastructure at the ART laboratory and general laboratory together as the mainstream laboratory such that all activities that complement each other take place in the same room. This could mean removing barriers such as walls and partitioning, using similar equipment in a common area, having one common sample collection/reception point and same patient flow methods. This can only be introduced where necessary and feasible considering laboratory space and work station. Laboratory process mapping is important and additional resources may be needed to achieve this. Space constraints is an inherent challenge in attaining physical integration.

#### Virtual/Service Integration

This focuses on combining each service element from the ART laboratory and integrating it into the mainstream laboratory services. Under virtual integration, a minimum package of laboratory service integration elements or key indicators were selected. The laboratory service integration elements minimum package include; a). Management Structure b). Trainings c). Equipment d). Equipment Maintenance e). Quality Management System f). Information Management System and g). Commodity Management.

### Study tools

**Figure 2:**
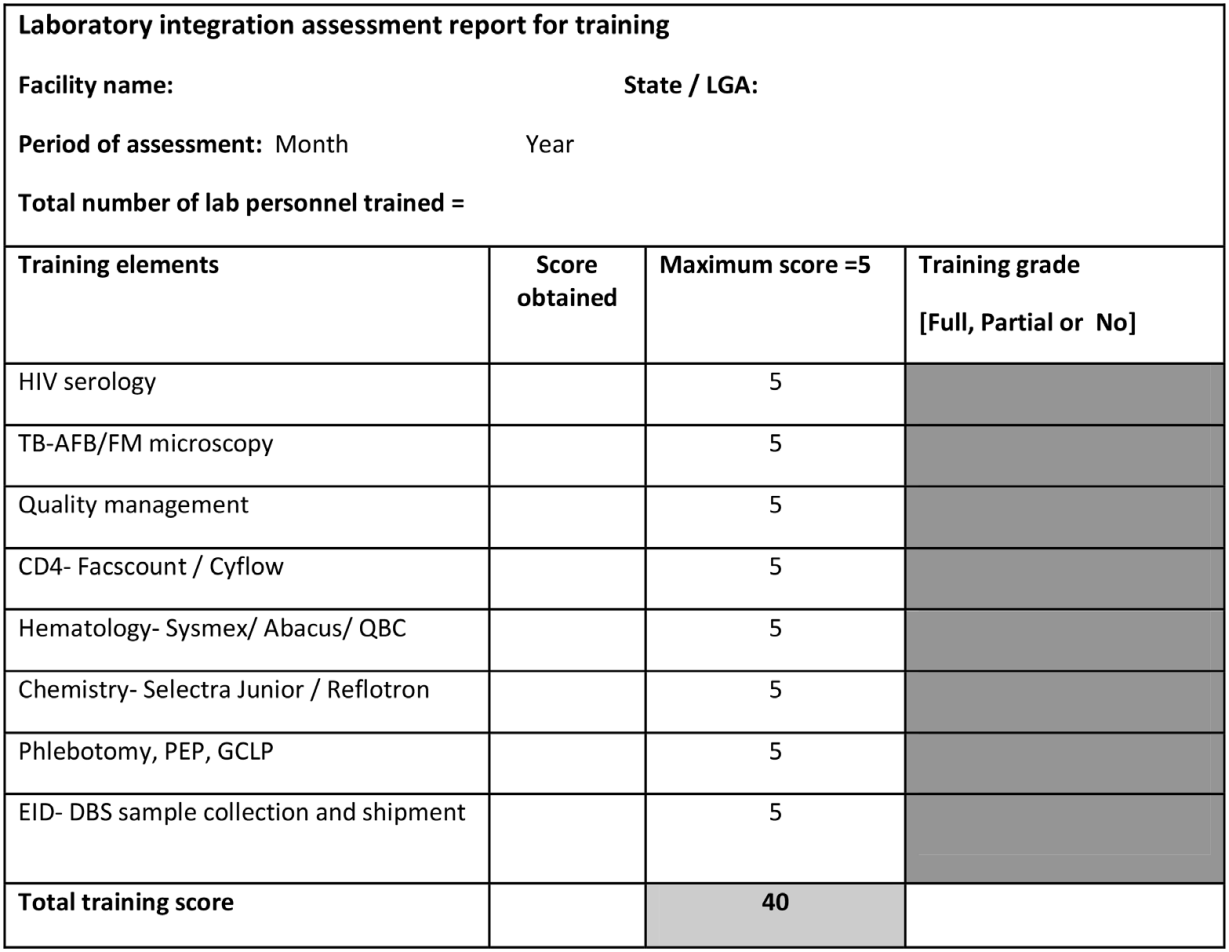
Summary of laboratory integration assessment report format for training. Scoring is based on the % of staff trained in each training element. 5 if ≥80%; 4 if between 60% and 79%; 3 if between 40% and 59%; 2 if between 20% and 39% and 0 if less <20%. Total Training grade should be filled from the training checklist portion. The total score obtained is added up across all training elements. The percentage score is got from the sum of scores obtained across all training elements expressed as a percentage of the total obtainable score of 40. This information is then captured in the Laboratory integration assessment report (figure 3) in the training row as follows; Rate as FULL (value 2) if ≥80%; Rate as Partial (value 1) if between 25% and 79%; and NO (value 0) if less <25%.

**Figure 3:**
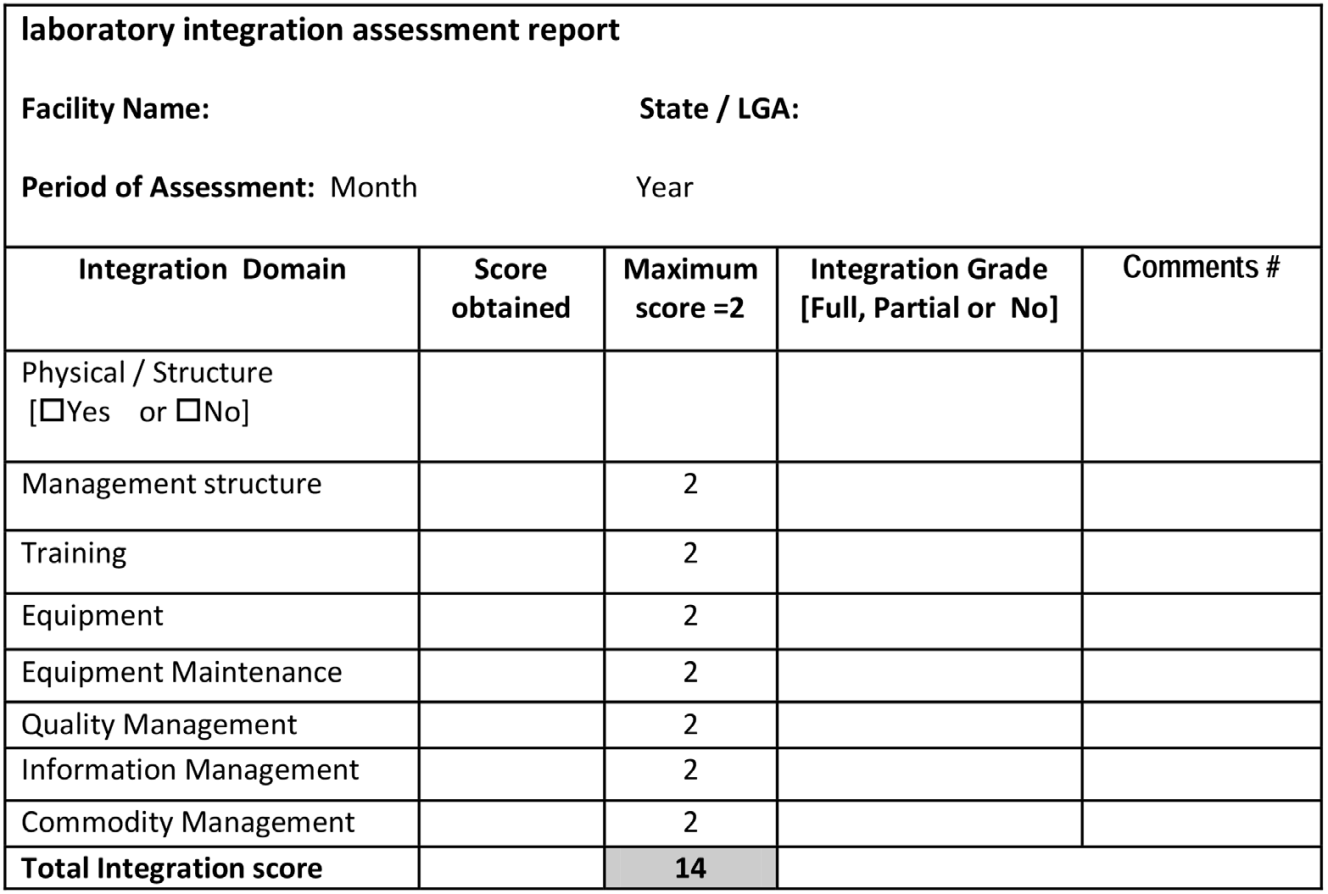
Summary of laboratory integration assessment report format. A Yes/No response was used to represent absence or presence of structural integration. Each virtual integration domain/service package rated as FULL receive a score of 2, PARTIAL receive a score of 1 while NO receive a score of 0 for that particular element. The percentage score is got from the sum of scores obtained in the various domains expressed as a percentage of the total obtainable score of 14. Following a defined composite score grading, ≥80% is considered as the facility having achieved FULL integration, and ≥25% to ≤79% is considered as the facility having achieved PARTIAL integration and while <25% is considered to be NO integration # noted gaps, recommendations and action plan. Note: Physical/structural domain is not included in the total integration score.

Tools were developed to quantitatively measure the level of service integration in the facility. The tools consist of facility summary sheet and integration checklist. The summary sheet is used to assess the various elements of integration activities within the facility following a guideline (Table 1). A copy of this sheet is kept in the facility and is available for review. The integration checklist is a MS Excel workbook consisting of two worksheets, a training summary report sheet (Figure 2) and the integration summary report sheet (Figure 3) containing information from the facility summary sheet. The integration summary sheet assesses the key parameters identified in the minimum package of virtual as well as physical laboratory integration. Level of integration was assessed on an ordinal scale (0 = no integration, 1 = partial integration, 2 = full integration) for each service package. Laboratory training element was separately captured in the training summary report before incorporating into the integration summary report as this element required careful measurement of different trainings delivered to laboratory personnel. A Yes/No response was used to represent structural integration. Detailed guide for grading and interpretation of findings is provided as footnotes in figures 2 and 3.

**Table 1:** Guidelines for scoring at assessment.

Integration domain	Summary of criteria to be regarded as full integration
Physical/structural	ART and mainstream facility laboratory are within the same structure without any physical demarcation.
Management	ART and mainstream facility laboratory services and personnel are coordinated and managed together under one leadership
Trainings	At least 80% of laboratory staff have been trained and have knowledge across various laboratory work stations to enhance staff rotation and reduce manpower challenges
Equipment	Specific assay equipment that complement one another are placed at the right workstation if possible, and/or utilized for similar function without separation for only ART purpose.
Equipment maintenance	ART and mainstream facility laboratory equipment have a common maintenance mechanism in place and managed under the same leadership.
Quality management	Quality indicators, policies, manual, SOP pertains to both ART and mainstream facility laboratory, quality control documents are available for all levels of testing. One quality manager oversees all quality matters
Information management	ART and mainstream facility laboratory have a central data collection and management system, and all tools are common.
Commodity management	ART and mainstream facility laboratory have a common logistics system whereby tools used for inventory management applies to both (CRRIF and Tally Card) and are available and in use for all items. Procurement of laboratory commodities are managed under the same system.

ART: antiretroviral treatment.

### Assessments

A pilot study was conducted in a secondary level facility (Maitama District Hospital, Abuja) to review the integration approaches, and the fitness of the tools to adequately capture and measure various elements of laboratory operational processes. Feedback was reviewed and adjustments were made. FHI360 laboratory staff and Government of Nigeria State Implementation Team (GON-SIT) were trained on the laboratory integration concept and how to use the tool to capture information on laboratory operational processes that does not involve human subjects.

The assessments were carried out at baseline and 3 months follow up period by a joint team of FHI360 laboratory staff and GON-SIT at the facilities starting from July 2012. The laboratory integration checklist was used to assess the level of integration. Data collected in the summary sheet was compiled at the state level and forwarded to FHI360 Country Office for further analysis.

### Ethical Clearance

FHI-360 Office of International Research Ethics (OIRE), North Carolina, USA, agreed with the submission that this project is not research and/or this research does not involve human subjects and waive the need for consent from participants.

### Data Management

The quarterly assessment data were imported into STATA version 10.0 (Stata Corporation, College Station, TX) for statistical analysis. Median differences between baseline and 3 months follow up assessment scores for each of the seven virtual integration elements as well as the composite total integration scores were tested using Mann Whitney U test, while paired sample t-test was used for the differences in mean percentage integration levels. Total integration score was obtained by summing up integration scores for all service elements (excluding structural integration), while percentage integration was computed by expressing the total integration score as a percentage of the maximum obtainable score of 14. Differences in proportions of structurally and virtually integrated laboratories at baseline and 3 months follow up were compared using Pearson’s Chi-squared test. A *p* value<0.05 was considered statistically significant for all analysis.

### Interventions

Between assessments the combined team of FHI360 technical staff and GON SIT carried out facility specific interventions to optimize the different laboratory integration domains in the facility. Key interventions include:

- Physical barriers like walls in some cases were collapsed. For example, in Maitama District Hospital, Abuja, machines were moved to a common area and alternate power source from generator connected to the entire laboratory. In some instances, one sample collection area was designated, which minimized the problem of stigma since there was no specific HIV phlebotomy section. Common patient registers were also encouraged.
- A common leadership management structure was established. The concept of HIV laboratory focal person was discontinued and the practice of having the head of the laboratory to manage all laboratory sections was instituted. General laboratory review meetings were also initiated. HIV laboratory clinic days were terminated. HIV and related tests were incorporated into the daily routine services.
- Trainings and mentorship were provided to all laboratory staff on HIV diagnosis and monitoring techniques, equipment use, laboratory quality management and other service components. Staff rotation was encouraged across all the various laboratory units, as laboratory staff were capacitated to perform diagnostic tests for diseases and conditions other than HIV, such as tuberculosis, malaria, fungal infections, bacterial infections etc.
- Equipment initially meant for ART services e.g. chemistry and hematology analyzers were now being used for the general public. Site engineers were trained to maintain all the equipment and in some cases the facility management extended maintenance service contracts to cover all equipment.
- With respect to quality management systems, a quality manager was nominated to oversee all sections of the laboratory. WHO/AFRO laboratory accreditation process [25] was extended to all the other laboratory units. Facility staff initially working in the HIV laboratory that had experience in WHO/AFRO laboratory accreditation process now serve as mentors to others, with a mandate to improve the entire laboratory system. Proficiency testing (PT) samples are analyzed generally or part of it analyzed at the other sections of the laboratory and the results compared and documented following feedback from the PT provider.
- For information management systems, computers, electronic and paper tools were distributed to all laboratory sections for documentation purposes and staff were trained on their use. Common patient registers were also implemented.
- Logistics management materials like Combined Request Report and Issue Form (CRRIF) were used across all sections as well as other tools for commodity forecast and quantification. A common commodity procurement system was instituted.

## Results

A total of 122 facilities were assessed. Nine facilities (7.4%) were primary level facilities, 104 (85.2%) were secondary level facilities and 9 (7.4%) were tertiary level facilities.

About 63 (51.6%) facilities had structural integration at baseline and 68 (55.7%) at 3 months follow up. The difference in proportions was not statistically significant (p = 0.5) (TABLE 2).

**Table 2:** Comparison of levels of laboratory service integration across domains at baseline and three months follow up.

Integration Domain	Baseline	Three Months Follow-up	P Value
**1. Structural/Physical Integration**			0.5
Integrated (Yes)	63 (51.6%)	68 (55.7%)	
Not integrated (No)	59 (48.4%)	54(44.3%)	
**2. Virtual/Service Integration**			
**a. Management Structure**			0.0002
Not integrated (0)	8 (6.6%)	5 (4.1%)	
Partially integrated (1)	12 (9.8%)	1 (0.8%)	
Fully integrated (2)	102 (83.6%)	116 (95.1%)	
**b. Trainings**			0.0000
Not integrated (0)	18 (14.7%)	13 (10.7%)	
Partially integrated (1)	84 (68.9%)	51 (41.8%)	
Fully integrated (2)	20 (16.4%)	58 (47.5%)	
**c. Equipment Use**			0.0000
Not integrated (0)	90 (73.8%)	48 (39.3%)	
Partially integrated (1)	19 (15.6%)	36 (29.5%)	
Fully integrated (2)	13 (10.6%)	38 (31.2%)	
**d. Equipment Maintenance**			0.0000
Not integrated (0)	102 (86.6%)	75 (61.5%)	
Partially integrated (1)	20 (16.4%)	45 (36.9%)	
Fully integrated (2)	0 (0.0%)	2 (1.6%)	
**e. Quality Management**			0.0000
Not integrated (0)	92 (75.4%)	56 (46.3%)	
Partially integrated (1)	27 (22.1%)	56 (46.3%)	
Fully integrated (2)	3 (2.5%)	9 (7.4%)	
**f. Information Management Systems**			0.0000
Not integrated (0)	79 (64.8%)	55 (45.1%)	
Partially integrated (1)	38 (31.2%)	60 (49.2%)	
Fully integrated (2)	5 (4.1%)	7 (5.7%)	
**g. Commodity Management**			0.0002
Not integrated (0)	79 (64.8%)	55 (45.0%)	
Partially integrated (1)	37 (30.3%)	64 (52.5%)	
Fully integrated (2)	6 (4.9%)	3 (2.5%)	
**Median Total Integration Score**	4 (IQR 3 to 5)	7 (IQR 4–9)	0.0000
**Mean Percentage Integration**	31.3±16.7%	45.6±20.2%	0.0000
**Status of Integration**			0.0000
Not integrated (<25%)	58 (47.5%)	19 (15.6%)	
Partially integrated (25%–79%)	64 (52.5%)	100 (82.0%)	
Fully integrated (≥80%)	0 (0.0%)	3 (2.4%)	

IQR: interquartile range.

*Each virtual integration domain/service package rated as FULL receive a score of 2, PARTIAL receive a score of 1 while NO receive a score of 0 for that particular element.*

*Following a defined composite score grading, ≥80% is considered as the facility having achieved FULL integration, and ≥25% to ≤79% is considered as the facility having achieved PARTIAL integration and while <25% is considered to be NO integration.*

There were statistically significant differences in integration levels between baseline and 3 months follow up period for all the laboratory elements considered under virtual integration (TABLE 2).

On a scale of 0 to 14, median total integration scores was 4 (interquartile range (IQR) 3 to 5) at baseline compared to 7 (IQR 4 to 9) at 3 months follow up (p = 0.0000) (TABLE 2). Mean percentage integration at baseline was 31.3±16.7% compared to 45.6±20.2% at 3 months follow up (p = 0.0000) (TABLE 2). Overall, 64 (52.5%) facilities laboratory systems were partially integrated, while no (0.0%) facility was fully integrated at baseline, compared to 100 (82.0%) partial integration and 3 (2.4%) full integration at 3 months follow up (p = 0000) (TABLE 2).

## Discussion

In this study, we demonstrated an innovative way of measuring integration of HIV related laboratory services into the mainstream laboratory operations. Core domains considered in the integration model include laboratory management structure, laboratory trainings, laboratory equipment usage, laboratory equipment maintenance, laboratory quality management systems, laboratory information management systems and laboratory commodity management. Some of these domains had been described as integral components of laboratory service integration [26], [27]. Our findings show statistically significant improvements in all integration service package domains between baseline and three month follow-up assessments. A composite measure of integration pooling together all domains into a model enables us to measure the status of integration of these laboratories per time. This enables us to define where each specific facility is on the integration continuum. Our study demonstrated that about a third of health facilities were able to move from “no integration” to “partial integration” in just three months of intervention, while three facilities attained full integration within the same period. This finding further lends credence to the assertion that integration of services is a continuum rather than a static state [28].

One major limitation of our methods is the exclusion of structural/physical integration in our model. This is premised on the fact that structural integration is dependent on availability of space and financial resources. Available resources at the onset suggested limited potential for the project to achieve any significant change in this domain. However, we assessed changes in level of structural integration between baseline and three month follow-up as an independent parameter. Expectedly, the changes were not statistically significant. A key strength of our method is that, we are able to demonstrate substantial improvement in integration of laboratory services in other core domains in spite of the difficulty with achieving structural integration. Although our current results show similar level of achievement on the laboratory commodity management domain, compared to some other domains, extra effort is required to address the associated challenges like commodity stock outs due to procurement flaws and late release of funds. Financial transparency is critical to this integrated laboratory model to avoid interrupted services to patients and ensure sustainability.

Our findings have the following implications for program implementation. Firstly, with the current emphasis of integration of donor funded activities into routine health services in order to engender sustainability, our methods demonstrate a viable option for quantification of levels of service integration achieved. Secondly, our study presents critical domains to address in enhancing project sustainability by increasing levels of service integration along a continuum over time. Lastly, our methods may be replicated in other vertical donor funded programs implemented at health facility level across different specialties to foster integration and entrench sustainability of project achievements. A probable follow-on study to this study is a longitudinal study measuring the changes in levels of integration over longer periods of project implementation and even beyond life of the project. Further research options include the adaptation of our methods in measuring levels of integration of other vertical donor funded programs into existing hospital services.

### Conclusion

To the best of our knowledge, this is the first study to document a method to quantitatively measure and classify the level of integration of HIV-related laboratory services into the mainstream laboratory in Africa. Our findings showed improvements in laboratory service integration and defined the status of each laboratory on the integration continuum. Whilst the rapid expansion of HIV laboratory services has left in its wake parallel structures, it has also provided the opportunity to use HIV-related laboratory strengthening as a means to improve the entire laboratory and health systems in general as experienced elsewhere [29], [30]. Our approach to measure and classify the level of laboratory service integration is feasible, affordable and scalable across supported sites. This method may be adopted in measuring levels of service integration of other vertical donor funded programs into the existing hospital services.
